# Information Dynamics of the Mother–Fetus System Using Kolmogorov–Sinai Entropy Derived from Heart Sounds: A Longitudinal Study from Early Pregnancy to Postpartum

**DOI:** 10.3390/e27090969

**Published:** 2025-09-17

**Authors:** Sayuri Ishiyama, Takashi Tahara, Hiroaki Iwanaga, Kazutomo Ohashi

**Affiliations:** 1 Maternal and Child Nursing, The Japanese Red Cross Kyushu International College of Nursing, Munakata 811-4157, Japan; 2 Institute for Basic Medical and Welfare Research, Fukuoka 810-0051, Japan; rsa05066@nifty.com (T.T.); a107163@gmail.com (H.I.); 3 Faculty of Global Nursing, Otemae University, Osaka 540-0008, Japan; ohashi@otemae.ac.jp

**Keywords:** chaos, complex systems, nonlinear dynamics, self-organization, Lyapunov exponent, Kolmogorov–Sinai entropy, information dynamics, heart sounds, maternal–fetal, heart sounds

## Abstract

Kolmogorov–Sinai (KS) entropy is an indicator of the chaotic behavior of entire systems from an information-theoretic viewpoint. Here, we used KS entropy values derived from the heart sounds of four fetus–mother pairs to identify the changes in fetal and maternal informational patterns during the four phases of pregnancy (early, mid, late, and postnatal). Time-series data of the heart sounds were reconstructed in a five-dimensional phase space to obtain the Lyapunov spectrum, and KS entropy was calculated. Statistical analyses were then conducted separately for the fetus and mother for the four phases of pregnancy. The fetal KS entropy significantly increased from early pregnancy to the postnatal period (0.054 ± 0.007 vs. 0.097 ± 0.007; *p* < 0.001), whereas the maternal KS entropy decreased in late pregnancy and then significantly increased after birth (0.098 ± 0.002 vs. 0.133 ± 0.003; *p* < 0.001). The increase in KS entropy with the course of fetal gestation reflects an increase in information generation and adaptive capacity during the developmental process. Thus, changes in maternal KS entropy play a dual role, temporarily enhancing physiological stability to support fetal development and helping to rebuild the mother’s own adaptive capacity in the postpartum period.

## 1. Introduction

The mother and fetus function as dynamically interacting systems, and understanding their interactions is of great importance in perinatal research. The human body is a complex mechanism comprising interconnected systems that do not function independently but interact dynamically and nonlinearly [1,2,3,4]. Conventional reductionist approaches attempt to understand this system by breaking it down into individual elements, but this often fails to capture the essence of the complex interactions [5,6,7,8]. As an alternative approach, the chaotic dynamical systems theory has been introduced, which is a framework for understanding the behavior of a system as a whole [9,10]. Definitions of chaos vary but are generally based on the fact that, although phenomena are deterministic, they are aperiodic and have initial value sensitivities [11,12]. Biological chaos has been studied for a variety of biological signals, including fingertip pulse waves [13], electroencephalography [14], and pupil diameter variability [15]. Tsuda et al.’s study of fingertip pulse waves showed that chaotic properties change dynamically with growth, aging, illness, and psychological states [13]. Also, in studies on infants, more complex chaotic patterns are observed in electroencephalography and heart rate variability as the child develops [16,17].

Our previous studies focused on dynamic interactions between the fetus and mother during pregnancy [18,19,20]. By simultaneously measuring heartbeats and analyzing the Lyapunov exponent, we found that the maximum Lyapunov exponent is positive and that the fetal and maternal systems exhibit deterministic chaos [18]. When analyzing the Lyapunov exponent, we also observed a distinct phase transition during gestation [19], at which time the fetal attractor changed from a near-structureless state to a more complex and chaotic structure as gestation progressed. In contrast, the mother’s attractor was chaotic and remained largely unchanged from early gestation until after birth. The difference in their patterns suggests that the fetus and mother have different chaotic dynamics and change differently over time [20].

In this exploratory study, rather than measuring local variations in the system, we instead focused on Kolmogorov–Sinai (KS) entropy, which is a measure that quantifies the rate of information production in a system as a whole. KS entropy is closely related to other nonlinear dynamical measures such as Lyapunov exponents, attractors, and Lyapunov dimensions, and it is especially useful for characterizing global system complexity in chaotic systems [12,21,22,23]. To the best of our knowledge, this is the first study to consider KS entropy from this perspective. We aimed to clarify changes in information generation and dynamic complexity from pregnancy to the postnatal period from the perspective of information dynamics. We performed a chaos analysis and KS entropy analysis on the heart-sound data of four pairs of fetuses (including newborns) and their mothers.

## 2. Materials and Methods

### 2.1. Subjects

This study was approved by the Research Ethics Committee of the Japanese Red Cross Kyushu International College of Nursing (Approval No. 12-06). All participants were given a verbal and written explanation of the purpose of the study, measurement procedures, voluntary participation, right to withdraw, potential benefits and risks, confidentiality of data, disclosure of results, and storage and destruction of any data collected. The pregnant women were specifically informed that if they felt unwell, they could immediately stop the heartbeat measurement and reaffirm their consent to continue. In addition, the participants were informed that they could withdraw from the study at any time by submitting a consent withdrawal form, and only those participants who gave written informed consent were allowed to continue in the study.

The subjects included five healthy women with uncomplicated singleton pregnancies and their fetuses, but one woman miscarried at 16 weeks of pregnancy, resulting in four subjects (A, B, C, and D) and their fetuses (including newborns). Two of the women were primiparous and two were multiparous, and they were aged 27 to 37 years. All pregnancies and deliveries proceeded without complications. To ensure the subjects were healthy and the pregnancies uncomplicated, the following inclusion criteria were applied: the absence of certain diseases or pregnancy complications (e.g., gestational hypertension, gestational diabetes), the absence of chronic diseases prior to conception (e.g., diabetes, hypertension, and heart disease), normal fetal development (e.g., no fetal growth retardation), and full-term delivery (at 37–41 weeks) by spontaneous or planned medical intervention of a fetus with normal birth weight and without congenital or chromosomal abnormalities or the need for any special medical intervention after birth. The data collection period was from March 2012 to March 2013.

### 2.2. Measurement Environment

All heart-sound measurements were performed in a quiet, private room with controlled environmental conditions (temperature: 23 °C, humidity: 50%). The measurement protocol was based on the method of Kisilevsky et al. [24]. Each mother was placed in a semi-seated resting position, and each measurement consisted of recording fetal and maternal heart sounds for a total of 5 min (1 min each, under five different situations of sound stimulation: no sound stimulation, when a recording of the mother’s voice was played, after the recording of the mother’s voice was played, when a recording of a stranger’s voice was played, and after the recording of a stranger’s voice was played). To ensure consistency across participants in all conditions, the recordings were of the individuals reading the same written text out loud (e.g., “How are you doing?”).

Voices were recorded and played back using a digital voice recorder (Voice Trek V-72, Olympus, Tokyo, Japan). The digital voice recorder was placed approximately 5 cm away from the mother’s abdomen, and the recordings were played back at a standard volume of 60 dB. Past studies of heart rate variability (HRV) analysis typically used data spanning 1 to 5 min as short-term HRV, and it has been reported that even 1-min data yields high sensitivity and reproducibility in terms of the Lyapunov exponent (LE) and entropy index [25,26]. In this study, based on prior research demonstrating that nonlinear indices such as the Lyapunov exponent and Kolmogorov–Sinai entropy can be reliably estimated from 1-min segments of cardiac time-series data [27,28], we adopted a 60-s interval for the analysis of heart-sound recordings. For postnatal recordings, the same procedure was applied by placing the voice recorder at a distance of 5 cm from the newborn’s head. This distance was chosen based on previous validation studies to minimize noise and ensure stable signal acquisition [19,20,29].

### 2.3. Heart-Sound Data Collection

Heart-sound data were obtained using two Doppler ultrasound devices (Doppler Fetal Detector FD-390, TOITU Medical Electronics Co., Ltd., Tokyo, Japan). The first probe of the device was placed in close contact with the mother’s heart to listen to the mother’s heart sounds, and the probe of the second device was placed in close contact with the mother’s abdominal wall to listen to the fetal heart sounds. Each Doppler device is equipped with an audio output jack. The analog heart-sound signals obtained were converted to digital signals at a sampling frequency of 1 kHz and 16 bits using an analog-to-digital converter (AI-1608AY-USB, CONTEC Co., Ltd., Osaka City, Osaka Prefecture, Japan), and the digital signals were saved on a computer using C Logger software Ver. 1.1 (CONTEC Co., Ltd., Osaka City, Osaka Prefecture, Japan).

Before data acquisition, the position of each probe was adjusted to ensure consistency in waveform amplitude and morphology. Data collection was initiated by pressing the recording switch, with a preset delay of 3 s after activation to allow for waveform stabilization before the start of digital recording. Each recording session lasted 60 s, yielding 60,000 data points per channel (sampling rate: 1000 Hz). At the end of each session, all waveforms were visually inspected to ensure data quality.

Heart-sound measurements were conducted longitudinally from early pregnancy through the postpartum period, with simultaneous recordings obtained from each mother–fetus pair.

### 2.4. Data Processing

Signal processing was performed as follows. Recordings were made in a quiet room, and the probe was firmly fixed to minimize motion artifacts (noise caused by movement of the probe or the subject). The sampling frequency was set according to the bandwidth of the electronic stethoscope. The 3M Littmann 3200 electronic stethoscope features multiple modes that emphasize the 20–500 Hz frequency range. In this study, it was determined that signals up to 500 Hz could be faithfully reproduced, and the sampling frequency was set to 1 kHz. After recording, a bandpass filter with a bandwidth of 20–500 Hz was applied digitally to remove low-frequency drift and high-frequency noise. Additionally, a low-pass filter (LPF) using a moving average method was applied to reduce baseline noise. Let s be the sampling time (seconds) and n be the number of moving average points. The approximate cutoff frequency is 1/(2ns). Furthermore, if this moving average is repeated *p* times, the equivalent cutoff frequency is $\frac{0.707^{\left(p-1\right)}}{2ns}$. In this study, n = 50 and *p* = 1 (with the moving average applied once), and the cutoff frequency was set to 10 Hz. The frequency of the component corresponding to the carrier wave of the heart-sound data was approximately 180 Hz (minimum 127 Hz, maximum 345 Hz) on average for all data. After removing the relevant high-frequency components, the data was judged to be suitable for analysis.

For artifact processing, all recorded data were visually inspected, and significant abnormal waveforms (peak shifts, excessive fluctuations, etc.) caused by external noise or body movements were excluded. The exclusion criteria were observed in cases of abrupt energy changes or continuous gaps, and for short-term gaps, linear interpolation was performed using the preceding and following waveforms. To confirm the reliability of the phonocardiographic measurements, ultrasound Doppler signals and electrocardiogram data were recorded simultaneously in adult male subjects, and agreement between the two signals was confirmed [20].

### 2.5. Lyapunov Exponent

The Lyapunov exponent (λ) [30] is a quantitative measure of the rate at which two initially close trajectories in a dynamical system diverge, and it is widely used as an indicator of chaos. If an infinitesimal perturbation δX(0) is applied to the initial condition, the separation between the two trajectories evolves as follows:

$$∥\delta X(t)∥\approx ∥\delta X(0)∥e^{\lambda t}$$

Here, *λ* denotes the Lyapunov exponent. More formally, the largest Lyapunov exponent is defined as

$$\lambda_{max}\underset{t\to \infty }{=\lim}\underset{∥\delta X(0)∥\to 0t}{\lim}\;\frac{1}{t}\ln\frac{∥\delta X(t)∥}{∥\delta X(0)∥}$$

A positive λ max indicates exponential sensitivity to initial conditions, signifying chaotic dynamics.

In our previous application of the Grassberger–Procaccia [31] and Sano–Sawada [32] methods to fetal and maternal heart-sound time-series data, we showed that fetal and maternal heart-sound time-series data are both five-dimensional [18]. Thus, the heart-sound time-series data were reconstructed in five-dimensional phase space, and the Lyapunov spectrum (λ_1_,λ_2_,λ_3_,λ_4_,λ_5_) was obtained every 10 s.

The Lyapunov spectrum refers to the set of all Lyapunov exponents corresponding to the independent directions in the phase space, rather than only the largest Lyapunov exponent. This provides a more comprehensive characterization of the system’s stability, mixing properties, and dimensional structure. In this study, five Lyapunov exponents (λ_1_,λ_2_,λ_3_,λ_4_,λ_5_), corresponding to the dimensionality of the reconstructed phase space, were computed, and their signs and magnitudes were analyzed to assess the dynamical properties of the system. In particular, since the sum of all positive Lyapunov exponents corresponds to the Kolmogorov–Sinai (KS) entropy, the KS entropy in this study was calculated using the positive components of λ_1_ and λ_2_, thereby quantifying the system’s rate of information production and chaotic complexity.

### 2.6. KS Entropy Analysis

Kolmogorov–Sinai (KS) entropy [21,22] is a measure that quantifies the rate of information production in dynamical systems [33], reflecting both the degree of chaos and the complexity of the system over time, as well as the magnitude of its unpredictability [34,35,36]. In addition, KS entropy serves as an index of the dynamic adaptability and self-organization of biological processes [2,37,38]. KS entropy is calculated as the sum of the system’s positive Lyapunov exponents (λ) [39].

$$hks=\sum_{\lambda_{1}>0}\lambda_{1}$$

Fetal and maternal data were categorized into four pregnancy phases (phase I: up to 20 weeks’ gestation; phase II: 21–26 weeks’ gestation; phase III: 27 weeks’ gestation to the prenatal period; and phase IV: postpartum). Within each phase, KS entropy values were pooled across all participants and experimental conditions without stratification by individual or stimulus presence. Classification was based on pregnancy phase rather than exact gestational age in weeks. The rationale for this phase division followed previous studies [20]. We then compared the KS entropy values of each phase using the linear mixed-model approach to examine the changes between phases. Subsequently, posterior comparisons were made after Tukey adjustment.

KS entropy was calculated using only valid segments when missing data occurred in some segments. To evaluate phase-dependent changes, a linear mixed-effects model was fitted with KS entropy as the dependent variable, phase (I–IV) as a fixed effect, and subject as a random intercept to account for within-subject correlation. Degrees of freedom were adjusted using the Kenward–Roger method. All statistical analyses were performed using R software (version 4.3.1; R Foundation for Statistical Computing, Vienna, Austria). Linear mixed-effects models were fitted using the lme4 package (version 1.1-34), and *p*-values were obtained with the Kenward–Roger approximation implemented in the lmerTest package (version 3.1-3).

## 3. Results

Table 1 summarizes the demographic characteristics of the participating mothers and newborns. There were two primiparous women and two multiparous women, all of whom had full-term pregnancies. All infants had a birth weight of 2500 g or more, with one male and three female infants. The Apgar score, an indicator of the health status of newborns, was normal.

From 11 weeks of pregnancy to 1 week postpartum, a total of 28 measurements were performed on the fetus and mother every 2–4 weeks (average 3.7 ± 1.3 weeks). The total number of segments for the Reaforov indices (λ_1_–λ_5_) of the fetus and mother was 4500. However, out of the total 4500 fetal segments, 4490 segments (99.8%) were calculable. Missing data were identified in 10 fetal segments (0.2%), limited to a 40–60-s interval during a single measurement at 26 weeks. Using the valid 0–40-s segments, the final sample size was the same as that of the mother, and the KS entropy sample sizes were 240 for Phase I, 210 for Phase II, 330 for Phase III, and 120 for Phase IV for both the fetus and the mother.

Table 2 shows the mean KS entropy values across phases for both fetuses and mothers. The results of the model diagnosis showed that the assumptions of normality and homoscedasticity of residuals were reasonably satisfied. The overall effect of phase was significant (Kenward–Roger method, *p* < 0.0001). Post hoc comparisons (Tukey-adjusted) revealed significant differences between all phases in both groups.

In fetuses, the largest difference was observed between phase I and phase IV (estimate = –0.042, *p* < 0.0001; Table 3). In mothers, KS entropy was slightly but significantly higher in phase II compared with phase I, significantly decreased from phase II to phase III (estimate = –0.017, *p* < 0.0001), and then markedly increased in phase IV compared with all other phases (estimate = –0.033, *p* < 0.0001; Table 3). The most pronounced difference was observed between phases III and IV, indicating a sharp increase in maternal KS entropy as delivery approached.

**Figure 1:**
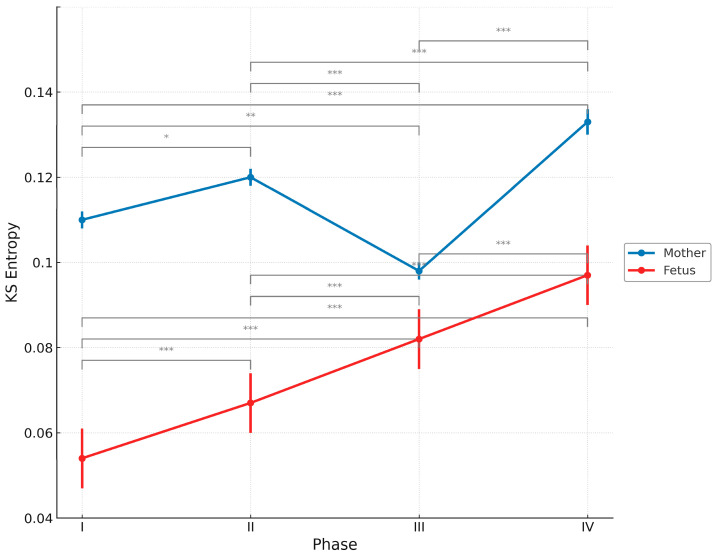
Changes in the average KS entropy and standard deviation across pregnancy phases for the fetus and mother. Phase I: ≤20 weeks of gestation; phase II: 21–26 weeks of gestation; phase III: 27–38 weeks of gestation; and phase IV: postnatal period. Note: The horizontal axis is divided into four phases for clarity of presentation; however, the actual durations differ among phases, as indicated. * *p* < 0.05, ** *p* < 0.01, and *** *p* < 0.001. Post hoc comparisons were performed using Tukey’s test.

Figure 1 shows how the mean and standard deviation of KS entropy in the fetus and mother changed during pregnancy. In the fetus, KS entropy increased in a stepwise manner from phase I (0.054 ± 0.007) to phase IV (0.097 ± 0.007), with significant differences between each phase (*p* < 0.001), suggesting that fetal heart-sound dynamics become increasingly complex as pregnancy progresses.

In contrast, the maternal KS entropy increased slightly (*p* < 0.05) from phase I (0.110 ± 0.002) to phase II (0.120 ± 0.002) and decreased from phase II to phase III (0.098 ± 0.002) (*p* < 0.0001). It then increased again during phase IV (0.133 ± 0.003). These changes may reflect a temporary adjustment mechanism in the mother in the last trimester of pregnancy and a physiological rebound after birth.

**Figure A1:**
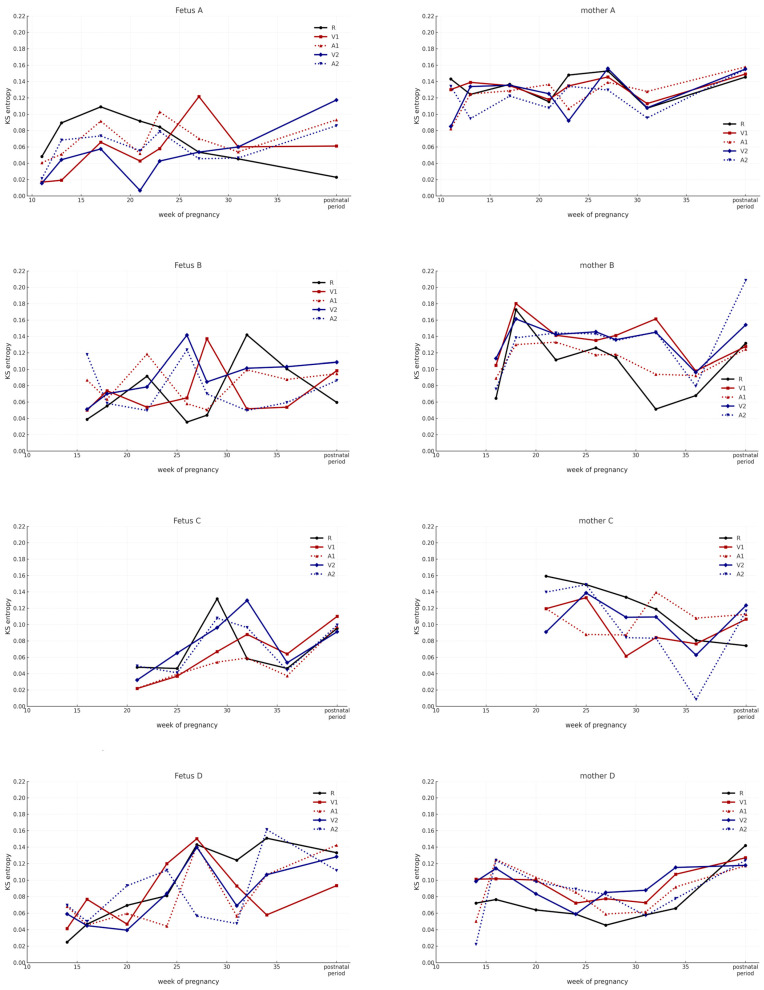
Longitudinal changes in KS entropy for each mother-fetus pair under five auditory stimulus conditions. Longitudinal changes in KS entropy for each auditory stimulus condition (R: resting; V1: mother’s voice; A1: after mother’s voice; V2: stranger’s voice; and A2: after stranger’s voice) in four mother-fetus pairs from early pregnancy to the postnatal period. Data points represent mean KS entropy values per measurement time for each condition. Measurements were repeated approximately every 2–4 weeks. Individual trajectories showed substantial variability between stimulus conditions and across participants. While certain time points revealed transient increases or decreases in KS entropy, no consistent trend across all pairs was observed within the same stimulus type. This variability suggests strong individual differences in maternal and fetal responses to sensory stimulation.

**Figure A2:**
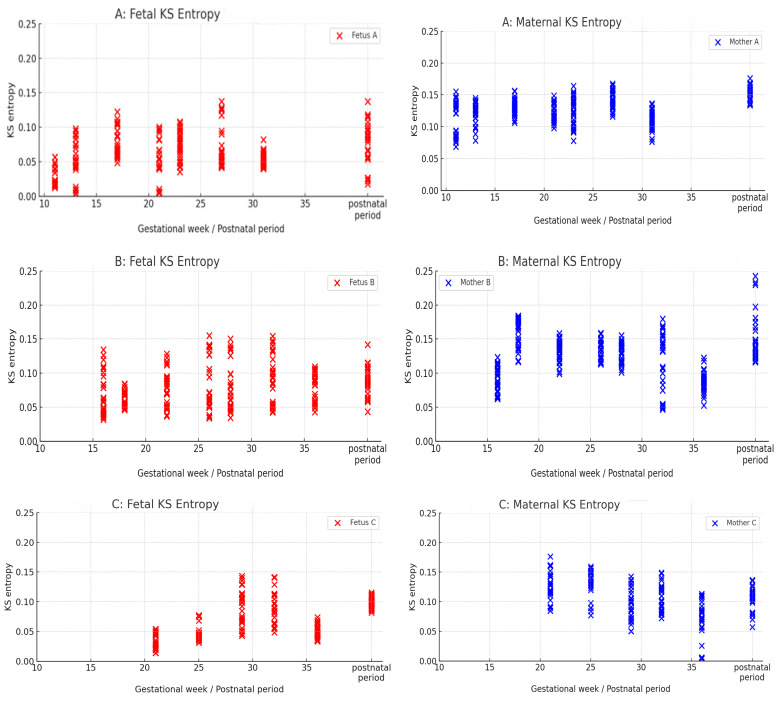

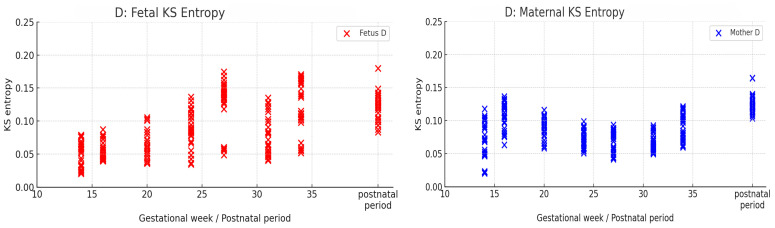
Longitudinal changes in KS entropy for each mother-fetus pair across gestational weeks and postnatal periods. Each panel shows scatter plots of KS entropy measured from maternal or fetal heart-sound time series, with each dot representing one measurement session. For each gestational week or postnatal period, 2–8 repeated measurements were obtained. Vertical spread at each time point reflects intra-week variability. Higher KS entropy indicates greater unpredictability of the heart rate signal. Fetus A (red): Shows a gradual increase in KS entropy toward late gestation, with a further rise postnatally. Mother A (blue): Relatively stable values during pregnancy, with a modest postnatal increase. Fetus B (red): Fluctuating values, with a mild upward trend toward term. Mother B (blue): Mid-gestation peak, followed by a decrease before term, then postnatal elevation. Fetus C (red): Low early-gestation values, increasing toward term. Mother C (blue): Stable mid-gestation values with postnatal rise. Fetus D (red): Marked late-gestation rise. Mother D (blue): Stable or slightly declining trend before a postnatal increase.

**Figure A3:**
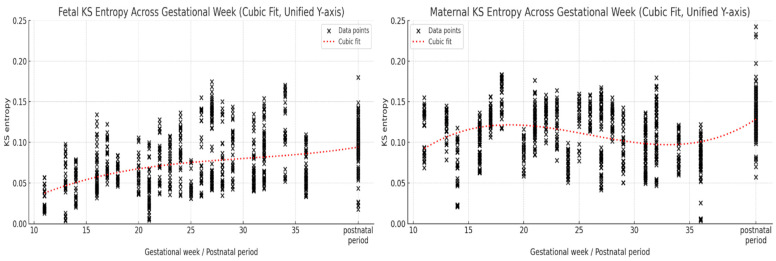
Overall cubic regression trends of KS entropy across gestational weeks for fetuses and mothers. Fetal KS entropy: N = 900 measurements from all fetuses; the cubic fit shows a gradual, consistent increase toward term and postnatal periods. Maternal KS entropy: N = 900 measurements from all mothers; the cubic fit (red dotted line) shows a modest early rise, a decline in late gestation, and a rebound postnatally.

In the initial analysis, KS entropy was calculated for each fetal–maternal pair (A–D) for each of the five sound stimuli (R, V1, A1, V2, and A2), according to the course of pregnancy (Appendix A Figure A1). Furthermore, we presented the KS entropy values for each fetal–maternal pair over the course of pregnancy for each case (Appendix A Figure A2). We then aggregated the data from all four cases by fetal and maternal group and plotted the changes over the course of pregnancy using regression curves (Appendix A Figure A3).

After adjusting for covariates (maternal age, fetal sex, and gestational age), the phase effect on KS entropy remained significant in both fetuses and mothers. Gestational age showed a small but significant effect in the maternal data, while other covariates were not significant (see Appendix A Table A1 and Table A2 for details).

## 4. Discussion

### 4.1. Significance of Heart-Sound Analysis

The heart sounds measured by the Doppler ultrasound method are not caused by the flow of blood but rather the movements of the heart muscle and the opening and closing of the heart valves. These movements are converted into audible signals by measuring the changes in ultrasound frequency corresponding to the temporal changes in the movement speed [40,41,42]. However, heart sounds and heart-sound waveforms do not merely represent the mechanical vibration of the heart. Heart sounds reflect nonlinear and dynamic biological responses, such as the activity of the autonomic nervous system and endocrine system, synchrony with respiration, physiological interactions such as blood flow, oxygen and nutrient exchange, and responses to emotions and external environmental stimuli [43,44,45,46]. Therefore, the heart-sound measurement observes how the physiological functions and information exchange between the fetus and the mother interact and change over time, rather than observing changes in each of them independently through the movement of the heart.

This can be interpreted as reflecting the “dynamic mutual adjustment field” created by both the fetus and the mother [47,48]. Additionally, the fetus is isolated from the external environment, and its heart sounds are one of the few continuous physiological signals that can be measured from the early stages of pregnancy until birth. Therefore, heart sounds reflect the fetal development process itself.

### 4.2. Importance of Aggregating Data

KS entropy was initially analyzed for each mother–fetus pair (A, B, C, and D) and for each of the five sound stimuli (no sound stimulation, when a recording of the mother’s voice was played, after the recording of the mother’s voice was played, when a recording of a stranger’s voice was played, and after the recording of the stranger’s voice was played).

However, as shown in Appendix A Figure A1, the time-series changes in response to stimulus conditions varied greatly among cases, and no specific consistent trend was observed. Therefore, in the main analysis of this study, KS entropy data were aggregated without distinguishing between cases or stimulus states and analyzed according to the phase of pregnancy (phase I: up to 20 weeks of pregnancy; phase II: 21–26 weeks of pregnancy; phase III: 27 weeks of pregnancy to before birth; and phase IV: after birth). This enabled a clearer understanding of the temporal changes in the global information-theoretic dynamics between mother and child, as shown in Figure 1. The fetal KS entropy was found to have significantly increased from early pregnancy to postpartum, while the maternal KS entropy was found to have decreased in late pregnancy and increased postpartum.

Two factors can be considered responsible for these results. The first is the effect of scaling, and the second is the effect of the central limit theorem. First, scaling is the idea that the behavior and structure of a system show different patterns depending on the scale of observation, such as the temporal, spatial, and aggregate unit observed [49]. In other words, the essence of scaling is that what is visible or invisible changes depending on which scale is looked at. For example, cellular functions may appear to consist of random movements when viewed at the molecular scale, but rules emerge when viewed at the tissue level. In the present study, by aggregating (scaling up) the mother–fetus pairs (cases) at an integrated level, it appears that characteristic changes in the entire system can be observed. In the present study, the dynamic changes observed in the whole system only emerged when data were aggregated across time (early to late gestation, postnatal), condition (5 stimulus types), and subject. This reflects a scaling perspective in which integration across multiple dimensions reveals the emergent properties of the system [50,51].

The second factor is the statistical effects of the randomness of the data. Statistically, data from different cases and conditions contain large variations, which can be regarded as random fluctuations, so it is difficult to see a constant trend. However, by aggregating the overall data, the central limit theorem [52,53,54,55] averages out the variations, and the KS entropy changes with the progress of pregnancy can be observed.

Thus, we considered that due to these two factors, the macroscopic trend of complexity possessed by the fetal–maternal system as a whole emerged as KS entropy, and that the characteristic development and features of the system as a whole became observable.

### 4.3. Temporal Changes in KS Entropy

An important aspect of this study’s results is the fact that we observed the first signs of human development. Fetal KS entropy steadily increased with advancing gestation from phase I to phase IV (0.054 ± 0.007 vs. 0.097 ± 0.007; *p* < 0.0001). This change is thought to be closely related to the maturation of the fetal autonomic and parasympathetic nervous systems. Van Leeuwen et al. have shown that the increased nonlinear variability in the fetal heart rate in late pregnancy reflects neural maturation and have reported that the complexity of the fetal heart rate variability increases with gestational weeks [56]. Furthermore, Signorini et al. have observed an increase in approximate entropy in fetal heart rate data through the use of linear and nonlinear analyses. This suggests that there is a possible link to the development of neural connections between the thalamus and cortex [57]. Thus, the increase in fetal KS entropy noted in this study may reflect the acquisition of more flexible and adaptive neuromodulatory functions mediated by the increased complexity of heart rate variability. In particular, the development of parasympathetic activity may lead to the emergence of subtle, short-term variations in the heart rate (high-frequency components) [58], resulting in the increased unpredictability of time-series data and, consequently, increased KS entropy. Furthermore, these results may reflect the increased complexity of information within the developing fetal system. From a dynamical systems perspective, such an increase may suggest increased adaptability and responsiveness to environmental and maternal stimuli [59]. As the fetal nervous, circulatory, and motor systems mature, the fetus exhibits more diverse responses resembling trial-and-error behavior [60], which can be interpreted as a primitive form of learning [61]. However, the results of the present study did not show that this change was only dependent on conditions such as vocal stimuli. This suggests that the stimulus condition is a short-term effect that elicits a response at that point in time but that the timing of gestation is a long-term effect that represents the underlying developmental state. In other words, it is not the stimulus condition that predominately determines the change in KS entropy but rather the temporal factor of the fetal stage of development.

In contrast, the mother’s KS entropy showed a short-term decrease in phase III before returning to an increase in phase IV (0.098 ± 0.002 vs. 0.133 ± 0.003; *p* < 0.0001) (Table 2 and Table 3, Figure 1). This is thought to be due to a temporary stabilization of the mother’s system in late pregnancy to support optimal fetal development [62]. This process potentially involves hormone regulation, autonomic nervous system regulation, and a reduction in physiological fluctuations, which contribute to the formation of a stable intrauterine environment [63,64]. This phenomenon reflects the reorganization of the internal environment and stabilization of physiological systems, especially in the pregnant mother; Shon et al. have reported an overall decrease in maternal heart rate variability in late pregnancy, which was attributed to a shift to sympathetic nervous system dominance and the suppression of parasympathetic activity [65]. Summer et al. have reported that, the mother’s heart rate variability shows a more stable pattern in late pregnancy, which they speculate is a physiological adaptation reflecting the preparation for labor [66]. Furthermore, DiPietro et al. have noted that changes in the hormonal environment (especially increases in estrogen and progesterone) during mid- to late pregnancy affect the autonomic nervous system and stress response system. The result is changes in the regulatory control of heart rate [67].

Thus, the decrease in the mother’s KS entropy during the late stages of pregnancy suggests that the variability of biological systems decreases, more efficient and stable control mechanisms are activated, and the body is transitioning to a preparatory state in anticipation of childbirth, which is a physiologically significant event. The decrease in maternal KS entropy during late pregnancy and its subsequent increase postpartum may reflect the reactivation of adaptive mechanisms in response to new interactions with the newborn following childbirth and physical separation from the infant. While not specifically addressing the postpartum period, Kaneko has proposed a theoretical framework suggesting that plasticity and robustness coexist within the hierarchical structure of biological systems [61]. This perspective is applicable to understanding the process of the structural and functional reorganization of the postpartum maternal body, and changes in maternal KS entropy from pregnancy to the postpartum period are inferred to reflect physiological stabilization and postnatal reorganization.

Furthermore, analysis accounting for potential covariates revealed that maternal age, fetal sex, and gestational age had no significant effects on fetal KS entropy, and the observed inter-observation variability was robust. Thus, fetal entropy changes are independent of extrinsic background factors and are thought to reflect intrinsic changes associated with developmental stages. On the other hand, in maternal KS entropy, gestational age remained a significant covariate, but phase effects were also independently significant. This suggests that maternal entropy changes reflect physiological adaptations associated with pregnancy progression while also undergoing adjustment effects specific to individual gestational ages.

The above findings support the notion that temporal changes in KS entropy in both the fetus and the mother are closely associated with pregnancy progression and are underpinned by distinct physiological mechanisms.

### 4.4. Fetal and Maternal Dynamics from an Information-Theoretic Perspective

The maternal KS entropy significantly decreased from phase II (0.120± 0.002) to phase III (0.098 ± 0.002, *p* < 0.0001) (Table 2 and Table 3, and Figure 1). From an information-theoretic perspective, this phenomenon can be interpreted as the mother temporarily suppressing her own information-generating capacity and chaotic unpredictability during late pregnancy to strengthen informational coupling with the fetus [1,61]. Such informational synchronization is particularly important in the later stages of pregnancy. This is because during this period, the autonomic nervous and sensorimotor systems of the fetus rapidly mature and begin to function as an autonomous information-generating system [60,68]. The mother provides a stable and consistent information environment to support this developmental process by providing a foundational site, so to speak. As a result, the mother’s own information dynamics temporarily subside. This process can be regarded as the optimization of the information environment [1,38].

Subsequently, the KS entropy of the mother increased significantly from phase III (0.098 ± 0.002) to phase IV (0.133 ± 0.003) (*p* < 0.0001) (Table 2 and Table 3, and Figure 1). In other words, after giving birth, mothers experience a reactivation of their own chaotic dynamics, returning from the temporary physiological stability of pregnancy and childbirth to the dynamic and flexible biological system they had before pregnancy. At the same time, mothers need to develop sensory, behavioral, and physiological adaptations to support their infants’ growth, and these changes can be understood as a reacquisition of complexity and flexibility [2,69,70].

The increase in the mother’s KS entropy observed during this period indicates that she is restoring her internal information-generating capacity and dynamic coordination in order to adapt to a diverse, chaotic, and unpredictable environment. This is consistent with postpartum endocrine and autonomic restructuring and responsive adjustments to emotional and social relationships [71], and it may be linked to the neurophysiological basis that supports the initiation of parenting behaviors [48].

On the other hand, the continuous increase in fetal KS entropy with the progression of pregnancy reflects the expansion of the information-generating capacity and the difficulty of chaotic prediction, as well as the progression of self-organization. The asymmetric changes in the mother’s temporary entropy decrease and the fetus’s entropy increase in phase III (Figure 1) suggest the maintenance of the total amount of information fluidity in the entire dual fetus–mother system. In other words, there is an informational complementarity at work between the two, which suggests the existence of a dynamic and adaptive information-exchange process between the mother and fetus.

The environmental discontinuity of childbirth causes a dramatic change in the information structure, and the mother again maximizes her own information-generating capacity and chaotic flexibility. This can be interpreted as a “return to information about one’s mother,” signifying a transition to a state in which the mother’s information network is once again open to both the outside world and the self. The present study analyzed KS entropy longitudinally based on mother and fetal heartbeat data from early pregnancy to the postnatal period. The observed changes in KS entropy indicate the existence of nonlinear and time-varying information dynamics between the fetus and mother and provide new insights into the understanding of the fetus–mother relationship as a chaotic adaptive network.

In the preceding framework of this study, we have positioned the fetus as a developing chaotic dynamic system and the mother as a mature chaotic system [19,20]. This relationship between mother and child can be understood not as a static, fixed structure but as a process of “chaotic coupling” that changes over time. That is, although the internal dynamics of each mother and child have different directions, they influence each other’s information generation and order formation through dynamic mutual coordination [48,61]. From this perspective, KS entropy can be positioned as an information-theoretic indicator of “coordinated development” in the mother–child system from gestation to birth. The temporary decrease in maternal entropy and the increase in fetal entropy indicate a dynamic process in which two chaotic systems with different phases are chronologically linked [68]. The “field” formed between the mother and fetus functions not as a mere physical space but as an integrated structure in which the flows of information and energy are intricately intertwined [37,47]. This “field” is believed to play a role in creating a new order in the information environment for both parties.

### 4.5. Clinical Significance and Application Potential

The KS entropy used in this study is an indicator that can non-invasively quantify the information-theoretic dynamics of the fetus and mother during pregnancy. The increase in fetal KS entropy observed in this study from early pregnancy to postpartum may reflect physiological development or neural maturation. However, at present, there is limited direct evidence regarding how these changes are associated with clinical outcomes such as developmental delays or neurodevelopmental disorders. Further longitudinal studies and analyses linking these changes to clinical outcomes are necessary to clarify their clinical significance.

Similarly, temporary decreases in maternal KS entropy during pregnancy and increases postpartum were observed, which may suggest adaptive physiological changes or stress responses. However, there is no established evidence linking these changes to postpartum physical or mental disorders or autonomic nervous system dysfunction. Therefore, these observations remain hypothetical findings that require further validation through future clinical studies.

### 4.6. Limitations and Future Directions

This study analyzed heart-sound time-series data obtained from four mother–infant pairs to clarify the characteristics of information-theoretic dynamics in fetuses and mothers, thereby presenting a new perspective on early human development. However, due to the small sample size (n = 4), caution is required when generalizing the findings. In the future, it will be necessary to apply nonlinear interdependence indices such as cross-entropy [72], transfer entropy [73], and synchronization index [74] to comprehensively evaluate the structural characteristics and interactions of the mother–infant system. In addition to a larger sample size, further the detailed investigations considering factors such as gestational age, maternal characteristics, and environmental factors, in addition to a larger sample size are required. Furthermore, longitudinal studies that track the same mother–infant pairs over longer period of time and at higher frequencies may reveal associations between chaotic characteristics in infancy and early childhood and neurodevelopmental and physiological adaptations. In addition, the fetuses participating in this study were three females and one male, and statistical comparisons were difficult due to the skewed sex ratio. However, no clear gender-specific effects were observed in covariance analyses. Nevertheless, previous studies have reported gender differences in heart rate variability and nonlinear entropy indices in children and newborns [75,76,77,78]. Therefore, the potential gender-specific effects on fetal KS entropy should be investigated in future studies with larger and more balanced samples.

Finally, KS entropy is a metric that can evaluate dynamic and adaptive complexity that is difficult to capture using traditional linear metrics, and it has the potential to provide a new analytical perspective in perinatal research. In the future, the application of nonlinear analysis could become a powerful tool for deepening our understanding of developmental individuality and mother-infant relationships.

## 5. Conclusions

This study represents the first attempt to conduct a longitudinal analysis of KS entropy based on maternal and fetal heart-sound data from early pregnancy through the postnatal period.

KS entropy in both the fetus and mother exhibits characteristic changes over the course of pregnancy. The increase in fetal KS entropy from early pregnancy through the postnatal period is thought to reflect information generation and improved adaptive capacity during the developmental process.

On the other hand, the decrease in maternal KS entropy during late pregnancy and its subsequent increase postpartum are speculated to serve a dual role: temporarily enhancing stability to support fetal development and establishing a foundational environment that enables this stability, followed by the reconstruction of the mother’s own adaptive capacity postpartum. Such growth, development, and adaptation in humans may be facilitated by the information-theoretic dynamics that exist within each individual and between the fetus and mother.

## Figures and Tables

**Table 1:** Characteristics of the four mother-fetus pairs.

Case	Mother’s Age	Parity	Method of Delivery	Gestational Age at Birth	Birth Weight (g)	Sex	Apgar Score
A	27	Primipara	Vaginal delivery	38 w 5 d	3038	male	9
B	29	Primipara	Planned caesarean section	38 w 4 d	2982	female	9
C	28	Multipara	Planned caesarean section	38 w 2 d	2874	female	8
D	37	Multipara	Vaginal delivery	41 w 1 d	2640	female	9

All four pairs had neither relevant medical history nor complications during pregnancy.

**Table 2:** Mean and standard deviation of KS entropy in fetus and mother across four phases.

Phase	Fetus (Mean ± SD)	Mother (Mean ± SD)
I	0.054 ± 0.007	0.110 ± 0.002
II	0.067 ± 0.007	0.120 ± 0.002
III	0.082 ± 0.007	0.098 ± 0.002
IV	0.097 ± 0.007	0.133 ± 0.003

KS entropy values are presented as mean ± standard deviation for each phase (I–IV) of pregnancy and early postpartum period.

**Table 3:** Pairwise comparisons of KS entropy between phases in fetus and mother.

Fetus						Mother				
Comparison	Estimate	Standard Error	df	t Value	*p*-Value	Estimate	Standard Error	df	t Value	*p*-Value
I vs. II	−0.013	0.003	894	−4.527	<0.0001	−0.007	0.003	895	−2.641	0.0418
I vs. III	−0.028	0.003	894	−10.686	<0.0001	+0.009	0.002	895	+3.975	0.0004
I vs. IV	−0.042	0.003	893	−12.425	<0.0001	−0.023	0.003	894	−7.643	<0.0001
II vs. III	−0.015	0.003	892	−5.620	<0.0001	+0.017	0.002	893	+6.897	<0.0001
II vs. IV	−0.029	0.003	891	−8.449	<0.0001	−0.017	0.003	893	−5.335	<0.0001
III vs. IV	−0.014	0.003	891	−4.401	<0.0001	−0.033	0.003	893	−11.483	<0.0001

Pairwise comparisons were conducted using Tukey’s method following a linear mixed-effects model.

## Data Availability

Raw data related to this study can be obtained from the corresponding author upon reasonable request.

## Abbreviations

The following abbreviations are used in this manuscript:

KS entropy	Kolmogorov–Sinai Entropy
λ	Lyapunov Exponent

## Appendix A

**Table A1:** Linear mixed-effects model results for fetal KS entropy (ANOVA with Satterthwaite approximation).

Effect	df	F Value	*p* Value	Significance
Phase	3, 890	5.35	0.0012	**
Year	1, 890	0.45	0.5021	n.s.
Sex	1, 890	0.22	0.6403	n.s.
Weeks	1, 890	1.12	0.2914	n.s.

Significance codes: ** *p* < 0.01.

**Table A2:** Linear mixed-effects model results for maternal KS entropy (ANOVA with Satterthwaite approximation).

Effect	df	F Value	*p* Value	Significance
Phase	3, 892	47.79	<0.0001	***
Year	1, 892	1.69	0.4172	n.s.
Sex	1, 892	0.06	0.8447	n.s.
Weeks	1, 892	5.65	0.0177	*

Significance codes: *** *p* < 0.001, * *p* < 0.05.
